# Roles for Treg Expansion and HMGB1 Signaling through the TLR1-2-6 Axis in Determining the Magnitude of the Antigen-Specific Immune Response to MVA85A

**DOI:** 10.1371/journal.pone.0067922

**Published:** 2013-07-03

**Authors:** Magali Matsumiya, Elena Stylianou, Kristin Griffiths, Zoe Lang, Joel Meyer, Stephanie A. Harris, Rosalind Rowland, Angela M. Minassian, Ansar A. Pathan, Helen Fletcher, Helen McShane

**Affiliations:** The Jenner Institute, University of Oxford, Oxford, United Kingdom; University of Padova, Medical School, Italy

## Abstract

A better understanding of the relationships between vaccine, immunogenicity and protection from disease would greatly facilitate vaccine development. Modified vaccinia virus Ankara expressing antigen 85A (MVA85A) is a novel tuberculosis vaccine candidate designed to enhance responses induced by BCG. Antigen-specific interferon-γ (IFN-γ) production is greatly enhanced by MVA85A, however the variability between healthy individuals is extensive. In this study we have sought to characterize the early changes in gene expression in humans following vaccination with MVA85A and relate these to long-term immunogenicity. Two days post-vaccination, MVA85A induces a strong interferon and inflammatory response. Separating volunteers into high and low responders on the basis of T cell responses to 85A peptides measured during the trial, an expansion of circulating CD4+ CD25+ Foxp3+ cells is seen in low but not high responders. Additionally, high levels of Toll-like Receptor (TLR) 1 on day of vaccination are associated with an increased response to antigen 85A. In a classification model, combined expression levels of TLR1, TICAM2 and CD14 on day of vaccination and CTLA4 and IL2Rα two days post-vaccination can classify high and low responders with over 80% accuracy. Furthermore, administering MVA85A in mice with anti-TLR2 antibodies may abrogate high responses, and neutralising antibodies to TLRs 1, 2 or 6 or HMGB1 decrease CXCL2 production during *in vitro* stimulation with MVA85A. HMGB1 is released into the supernatant following atimulation with MVA85A and we propose this signal may be the trigger activating the TLR pathway. This study suggests an important role for an endogenous ligand in innate sensing of MVA and demonstrates the importance of pattern recognition receptors and regulatory T cell responses in determining the magnitude of the antigen specific immune response to vaccination with MVA85A in humans.

## Introduction

Tuberculosis (TB) remains a major global health issue, with an estimated 8.7 million cases and 1.4 million deaths in 2011 [1]. BCG, the only licensed vaccine against TB, shows only partial, variable efficacy against pulmonary TB [2–4]. Twelve candidate vaccines are currently in clinical trials [5] and results of the first efficacy trial of a new vaccine against *Mycobacterium tuberculosis* (*M.tb*), in which Modified Vaccinia virus Ankara expressing antigen 85A (MVA85A) was given to BCG-vaccinated South African infants, have recently been reported [6]. Although MVA85A did not confer improved protection to TB compared to BCG alone in this setting, further analysis of samples collected will provide a valuable opportunity to investigate the immune basis of protection against TB.

Efforts to produce T cell inducing vaccines against diseases such as TB, HIV and malaria, have made use of viral vectors as antigen delivery systems to enhance the immune response to the antigen of interest. Vaccine candidates are selected on the basis of safety, efficacy in preclinical animal disease models and the ability of the vaccine to induce the secretion of interferon-γ (IFN-γ) by antigen-specific CD4+ and CD8+ T-cells [7–9]. The secretion of cytokines by stimulated cells, particularly IFN-γ, remains the primary gauge of vaccine-induced adaptive immune responses in both animals and humans [10]. IFN-γ is essential for protection against TB and, although it is not a correlate of protection, it correlates well with other Th1 functions and is the cytokine that gives the most robust measure of response to vaccination [11,12]. Therefore it is of paramount importance to understand the mechanisms by which different vaccines induce this immune response, as well as understanding the basis of a protective immune response to the diseases for which the vaccines are being tested.

Attenuated poxviruses have been widely used in vaccine development; MVA for example has been tested in clinical trials against malaria, TB, HIV, influenza and a variety of cancers [13–18]. There is now interest in determining how MVA initiates the immune response. The induction of apoptosis by MVA is well documented [19–21] and recent work has shown that deletion of the anti-apoptotic gene F1L from the genome of MVA leads to increased immune responses in mice, suggesting apoptosis plays an important role in the induction of immunity to MVA [22]. Additionally, Delaloye et al. have shown the importance of Toll-Like Receptors 2 and 6 (TLR2-6), melanoma differentiation-associated gene 5 protein (MDA-5) and the NALP3 inflammasome in immune sensing of MVA [23], although no ligand for this association has yet been characterized. HMGB1, a nuclear protein released during cell death acts as a danger associated molecular pattern (DAMP) and can bind to several TLR receptors. It has been implicated in the immune response to several viruses and has been proposed as the elusive TLR2 ligand during MVA and vaccinia infection.

The field of vaccinology has seen, through seminal work using the yellow fever vaccine YF-17D as a model, that systems biology can be a powerful tool in identifying the mechanisms by which an immune response to vaccination develops [24,25]. In this study we describe the innate immune response to MVA85A in BCG-vaccinated volunteers in the UK and use the data to investigate the links between early changes in gene expression and IFN-γ ELISpot responses measured at the time of the trial. We are not able to postulate on the relevance of this to protection, as correlates of protection remain an elusive goal. However, the *ex-vivo* IFN-γ ELISpot is a good measure of vaccine “take” and correlates with many aspects of Th1 type immunity. It has been used in multiple studies across different diseases to assess vaccine immunogenicity, although it is not a correlate of protection in any of these diseases. In the case of tuberculosis, IFN-γ is also known to be necessary, though insufficient, for protection. Understanding the mechanisms underlying the immune response to vaccination is an important goal that complements but is separate from studies examining the basis of protective immunity. MVA85A is designed to augment the T cell responses induced by BCG through expansion of antigen 85A-specific T cells, and the immune response to MVA85A has been studied using the *ex-vivo* IFN-γ ELISpot in multiple populations. This work shows the majority of the antigen-specific response to MVA85A in BCG-vaccinated individuals is mediated by CD4+ T cells, peaks around 7 days after vaccination and is maintained at a level above baseline for at least 6 months [15,26–29]. Here we find that differences in the regulatory response between volunteers two days after vaccination are important in determining the magnitude of the ELISpot response, as is signaling through the TLR2 axis. Low responders express higher levels of Treg markers including CTLA4, IL2RΑ and STAT5B pre- and 2 days post-vaccination and show an expansion of the CD4+ CD25+ Foxp3+ Treg population in the first week post-vaccination. Additionally, blocking TLR2 signalling decreases the response to MVA85A and this is likely mediated by the danger associated molecular pattern (DAMP) HMGB1, released from dying cells infected by MVA and signaling through TLR2-6 receptors.

## Results

### Innate Immune responses to MVA85A

Samples used in this study were taken from a trial of 24 BCG-primed healthy adults from the UK, vaccinated either intradermally (ID) or intramuscularly (IM) with 1x 10^8^ plaque-forming units (pfu) MVA85A. Full details of the trial have been published [30]. Peripheral blood mononuclear cells (PBMC) from volunteers were cryopreserved on day of vaccination and at the following timepoints: day 2 and weeks 1, 2, 4 and 12 post-vaccination. IFN-γ ELISpots to antigen 85A peptide pools were done on fresh PBMC at each time point except day 2 (summary plot shown in Figure S1). In this study unstimulated PBMC from day of vaccination (day 0) and two and seven days later (day 2, day 7) were thawed and lysed for gene expression analysis on Illumina microarrays.

The median IFN-γ ELISpot responses to 85A peptides were not significantly different between the IM and ID groups at any time point [30] and, from a filtered list of 22,000 genes, no genes were differentially expressed between the two groups at any time point. Both groups were therefore combined for all subsequent analyses.

**Figure 1:**
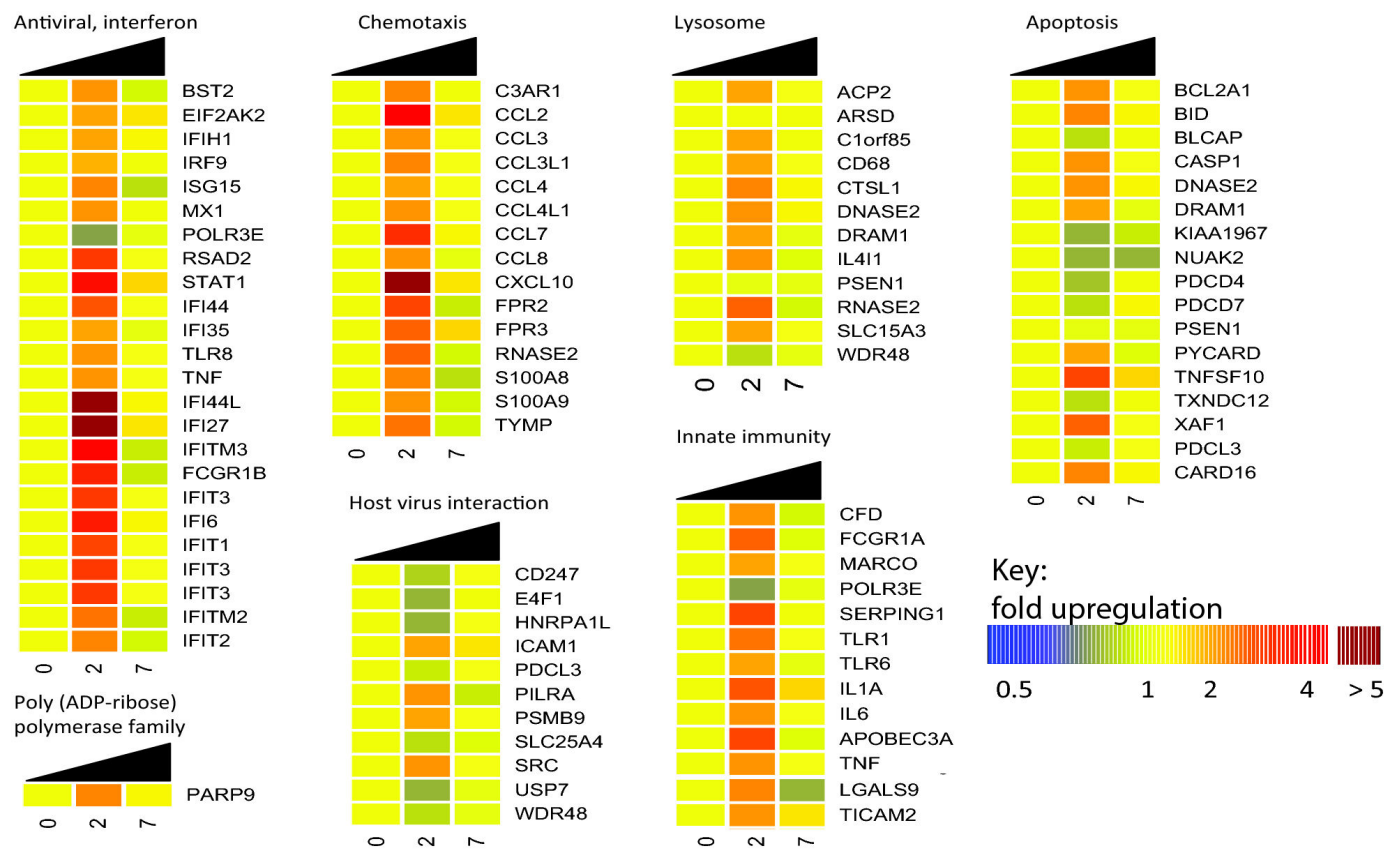
Transcriptional response to MVA85A. Heat map showing changes fold change in gene expression of common genes 2 and 7 days following MVA85A vaccination. The genes are sorted into categories based on DAVID Bioinformatics Database gene descriptions. The heat map colours show the average expression across the 24 volunteers at each time point (given in days at the bottom of each column).

Comparing two days after vaccination to baseline, 1100 genes were differentially expressed (false discovery rate <0.05). Heatmaps showing separation of individual volunteers using these genes is shown in Figure S2. Analysis of these genes using Gene Set Enrichment Analysis [31–33] (GSEA) and the Database for Annotation, Visualisation and Integrated Discovery [34] (DAVID) showed an enrichment for gene ontology (GO) terms and gene sets relating to an inflammatory response, positive regulation of the immune response, lysosome and apoptosis (Table 1). By day 7, gene expression had returned almost completely to baseline (Figure 1). The greatest change in gene expression was seen in the interferon-induced chemokine CXCL10 (IP-10) with a fold change of 8.5 on day 2 compared to day 0.

**Table 1:** Genes upregulated 2 days post vaccination with MVA85A compared to baseline in healthy volunteers.

Cluster	Annotation term^a^	Adjusted
(enrichment score)		p-value
Defense Response (9)	Defense response	3.00E-10
	Inflammatory response	9.30E-06
Lysosome (6.52)	Lysosome	3.70E-06
NF-κB Signalling (3.27)	Positive regulation of I-κB kinase/NF-κB cascade	8.90E-03
Immunoglobulin	IgG binding	4.30E-04
Binding (3.25)	Immunoglobulin binding	4.40E-04
Immune Response	Positive regulation of immune response	3.00E-05
(3.24)	Lymphocyte mediated immunity	7.50E-04
	Innate immunity	8.40E-04
Apoptosis (3.19)	Regulation of programmed cell death	1.30E-02
	Regulation of apoptosis	1.60E-02
Chemotaxis (2.58)	Chemotaxis	2.40E-03

a Representative annotation terms and their p-value for each annotation cluster.

We compared this gene signature with changes in gene expression of PBMC stimulated six hours *in vitro* with either media alone, antigen 85A, wild type MVA (MVAwt) or MVA85A. The PBMC used were from day 0 samples (prior to vaccination with MVA85A) from the above study. The four volunteers included were randomly chosen from both low and high responders to MVA85A (based on previously performed ELISpot assays). Of the 1100 genes differentially expressed on day 2 in the volunteers, 50% were also differentially expressed in the cells stimulated *in vitro*. These genes were enriched for GO terms including phagocytosis, chemotaxis and the inflammatory response. The 550 genes upregulated *in vivo* but not *in vitro* were enriched for genes involved in the innate immune response. A further 4000 genes were differentially expressed in MVA85A stimulated PBMC compared to the controls, including genes involved in the lysosome, inflammation, apoptosis, cytokine-cytokine interactions and leukocyte migration.

Infection with either MVA85A or MVAwt leads to differential expression of around 5000 genes compared to the control; of these, over 3000 were modulated by both MVAs. 176 genes show significant differential expression when PBMC were stimulated with MVA85A compared to MVAwt. Around 30 genes were significantly upregulated compared to the negative media only control when stimulated with any of antigen 85A, MVA wild type or MVA85A. A list of these is included in table S1.

### High and low responders show differences in gene expression pre- and post-vaccination

#### Regulatory T cells increase in low responders following vaccination

In order to gain an insight into the mechanisms associated with differences in ELIspot responses between volunteers, we divided the 24 volunteers into “high” or “low” responders based on an area under the curve analysis for IFN-γ ELISpot responses to 66 overlapping peptides covering antigen 85A [30]. AUC correlated with immunogenicity at each time point post-vaccination (r>0.77, p<0.0001 for all comparisons) and was used to avoid problems associated with multiple testing. Volunteers with a response greater than the median AUC were classified as high responders. Additionally, pre-vaccination responses to purified protein derivative from *M.tb* (PPD-T) or to antigen 85A do not correlate with post-vaccination ELISpot responses to 85A peptides (r=0.16, p=0.43 to PPD-T; r=0.22, p=0.31 to 85A peptide pools), none of the volunteers had been previously vaccinated with a recombinant MVA and almost all were too young to have been vaccinated against smallpox which ceased to be part of the routine vaccination schedule in the UK in the early 1970s (age range 18-55 years, median 27.5 years).

Two days after vaccination, 176 genes were differentially expressed between high and low responders. This list of genes was enriched for GO terms associated with regulation of T-cell activation and co-stimulation signal (DAVID, fdr<0.05). The genes contributing to the enrichment of these clusters, which are more highly expressed in low responders, are shown in table 2. Although the fold change is low, the enrichment of genes associated with regulation of T cells suggests this change is of biological relevance. Furthermore, mRNA of several genes correlated directly with the IFN-γ ELISpot responses to vaccination.

**Table 2:** Differentially expressed genes between high and low responders, two days after vaccination.

SYMBOL	Description	Fold Change	adj. p-value
CTLA4	Cytotoxic T lymphocyte antigen 4	1.77	0.037
CD5	T cell surface antigen	1.62	0.032
IL2RΑ (CD25)	IL2 receptor, α subunit	1.58	0.043
TRAT1	TCR associated adaptor molecule	1.48	0.03
STAT5B	T cell transcription factor	1.48	0.043
CD28	co-stimulatory molecule	1.43	0.038
CD3D	T-cell receptor, δ subunit	1.41	0.025
ITK	IL-2 inducible T cell kinase	1.4	0.048
CD2	T cell surface antigen	1.34	0.047
DNAJA3	DnaJ (Hsp40) homologue	1.3	0.034

**Figure 2:**
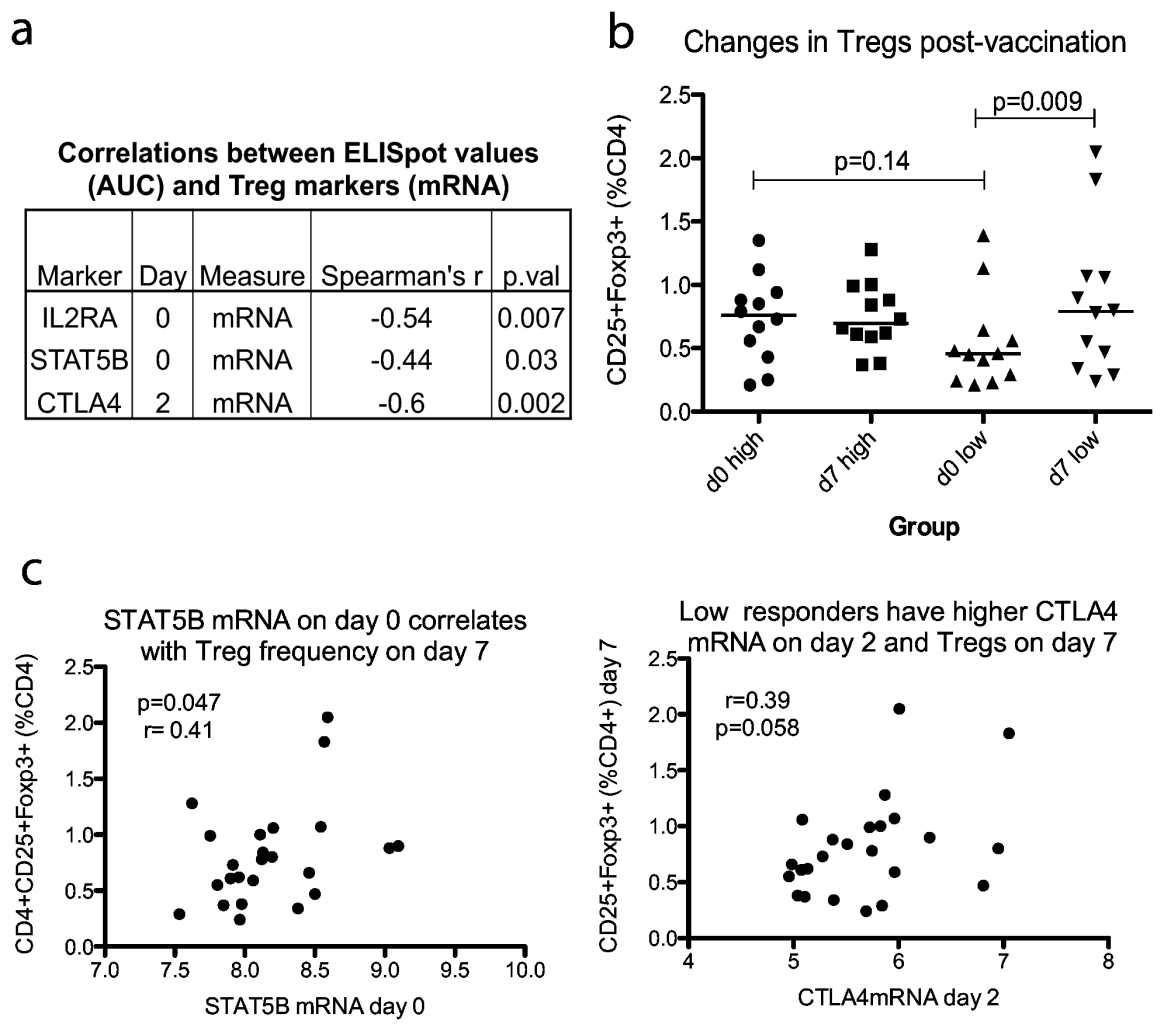
Low responders make a stronger regulatory response to vaccination with MVA85A. To further explore the relationship between Treg marlers and IFN-γ ELISpot response identified through the microarray, flow cytometry of Treg markers was done of cells from the same volunteers taken on day of vaccination (day 0) and 1 week later (day 7). - A. mRNA levels as measured by microarray for several Treg markers show an inverse correlation with the IFN-γ ELISpot response. B. The Treg population (CD4+ CD25+ Foxp3+) is increased in low but not high responders during the first week following vaccination (paired t-test, p=0.009) C. STAT5B expression on day 0 correlates with frequency of Tregs (CD25+ Foxp3+, % CD4+) and there is a trend between expression of CTLA4 on day 2 and Tregs on day 7. Treg absolute counts: mean: 1687, median: 1631, interquartile range: 740-2882.

To investigate this association further, we then examined whether increased expression of genes associated with a regulatory response 2 days after vaccination is reflected in the regulatory T cell (Treg) compartment. The number of Tregs (CD4+ CD25+ Foxp3+) in unstimulated PBMC from day of vaccination and 7 days later were quantified by flow cytometry. In high responders, the number of Tregs (as a percentage of CD4+ cells) remained constant pre- and post vaccination, however in low responders it increased between day 0 and day 7 (Figure 2a, p = 0.009). The expression of STAT5B mRNA on day 0 correlated with the percentage of Tregs seen in PBMC on day 7 (p=0.41, r=0.047). There was also a weak association between CTLA4 mRNA on day 2 and percentage of Tregs seen in PBMC on day 7 although the correlation did not reach significance (Figure 2b, r_s_ = 0.39p=0.058).

#### TLR1, TICAM2 and CD14 are more highly expressed in high responders on day of vaccination

**Figure 3:**
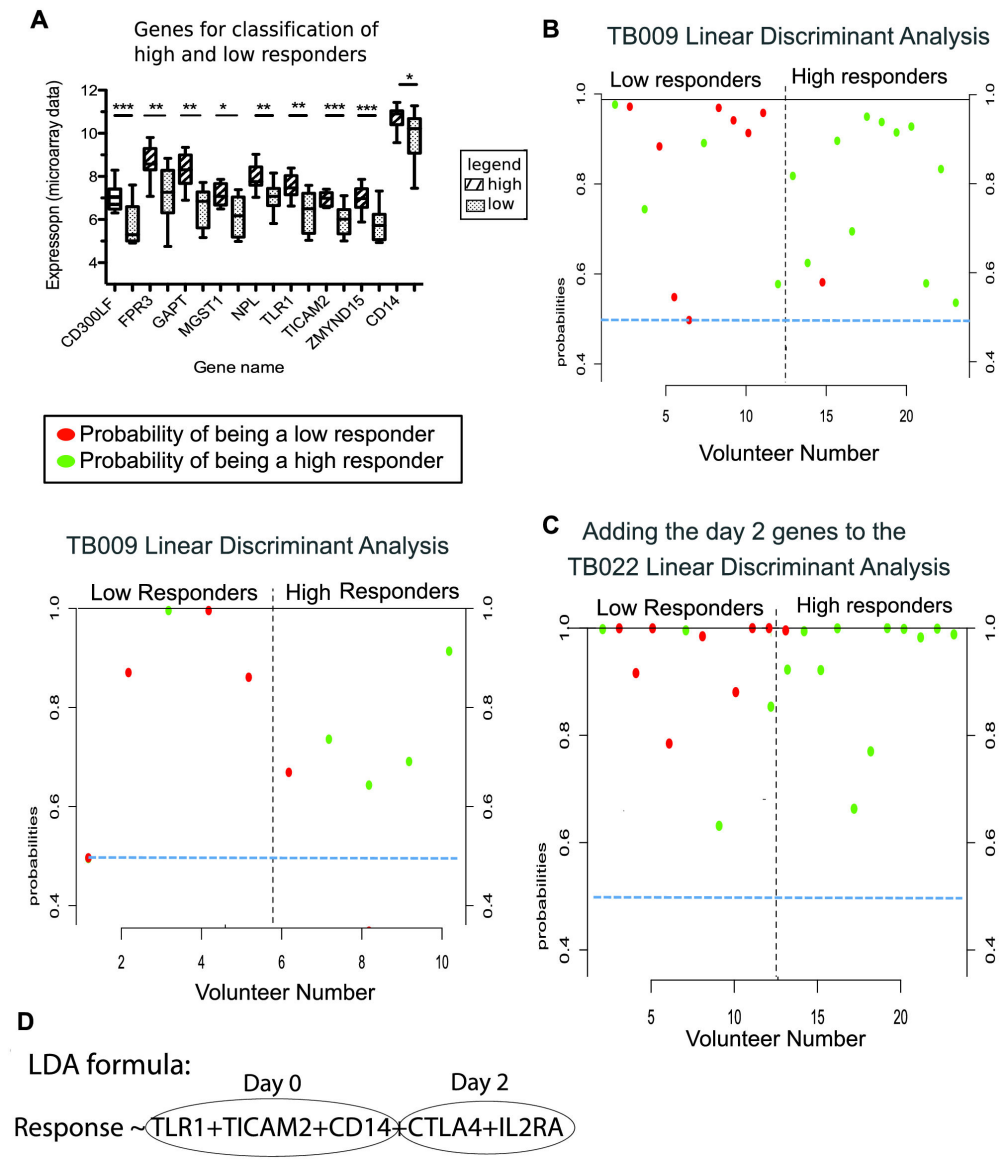
Expression of genes on day of vaccination and 2 days later can classify high and low responders. Cohorts: TB022: 24 volunteers used in the original microarray analysis. TB009: 10 volunteers from a previous trial of MVA85A who received an equivalent dose used in the qPCR validation. A. Exploratory list of 9 genes obtained from the prediction analysis done on the microarray data from TB022 B. Probability plots of linear discriminant analysis classification outcomes of high and low responders in trials TB022 and TB009 from expression of TLR1, TICAM2 and CD14 on day of vaccination. C. Probability plot of LDA classification outcome of TB022 volunteers when CTLA4 and IL2RΑ expression on day 2 are added to the model (Other genes, eg CD5 and STAT5B were removed as they did not add predictive value to the model due to co-linearity with other genes). D. Hypothesis resulting from LDA analyses.

On day of vaccination (day 0), 400 genes were differentially expressed between high and low responders however these were not enriched for any GO terms. We analysed these genes using pamR (prediction analysis for microarray) [35] and identified nine genes that could classify the 24 volunteers as high or low responders with a cross validation accuracy of 87% (Figure 3a; **GAPT**: GRB2-binding adaptor protein, transmembrane; **ZMYND15**: zinc finger, MYND-type containing 15; **MGST1**: microsomal glutathione S-transferase 1; **CD300LF**: CD300 molecule-like family member f; **NPL**: N-acetylneuraminate pyruvate lyase; **FPR3**: formyl peptide receptor 3; **TLR1**: toll-like receptor 1 and **TICAM2**: toll-like receptor adaptor molecule 2).

To determine the reproducibility of this finding, we used 10 samples from a previous trial in which the volunteers were vaccinated ID with an equivalent dose of MVA85A [36]. We measured the expression of the nine genes by qPCR and again classified the volunteers as high or low responders based on the median AUC value for their ELISpot responses (in both trials, the medians were 41000 spots. time/million PBMC). We then performed a univariate logistic regression for each gene and, as the sample size was small, retained all genes with moderate discriminatory power (p≤0.2).

The three genes retained were: TLR1, CD14 and TICAM2. While CD14 expression correlates with AUC (p=0.006, r=0.3), monocyte levels (as determined by full blood counts) do not (p=0.9, r=0) and therefore CD14 expression is not simply a reflection of the number of circulating monocytes. Using linear discriminant analysis (LDA), the mRNA levels of TLR1, TICAM2 and CD14 on day of vaccination could classify individuals as high or low responders with a cross-validation accuracy of 80%. Doing the same LDA analysis on the original 24 samples, using microarray values for the expression levels of the same three genes, the cross validation accuracy was 79% (Figure 3b).

Although no discriminatory gene set was found based on gene expression 2 days after vaccination, adding CTLA4 and IL2RΑ (CD25) to the LDA model developed with the three innate sensor genes refined the model and increased its cross validation accuracy to 88% (Figure 3c, d). Unfortunately we were not able to validate this finding on the second trial, as day 2 samples were not available.

#### TLR1 expression on day of vaccination correlates with ELISpot responses

**Figure 4:**
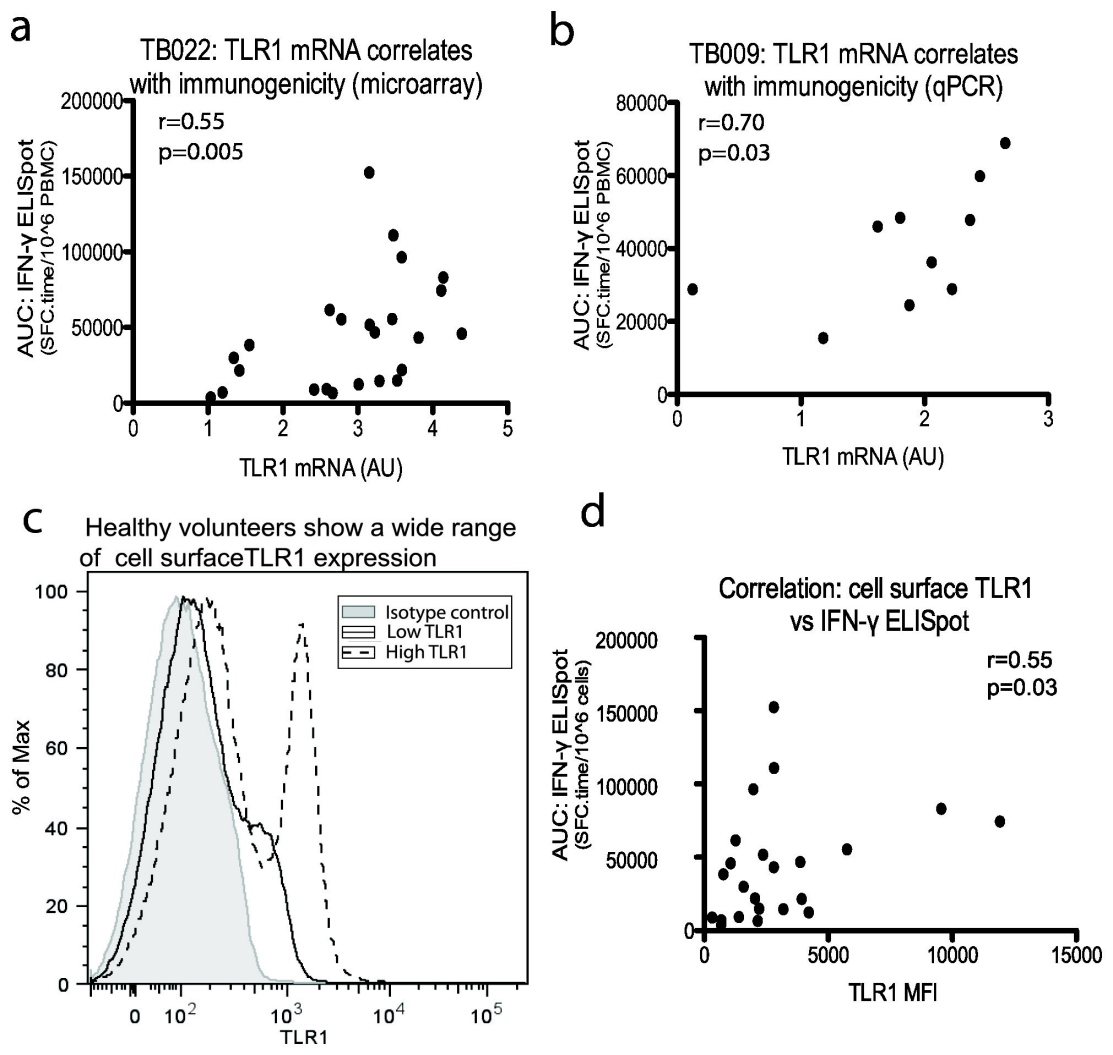
TLR1 levels on day of vaccination correlate with immunogenicity. A. Levels of TLR1 mRNA correlated with long term immunogenicity to MVA85A in TB022 (Spearman’s rank correlation). B. Levels of TLR1 mRNA correlated with long term immunogenicity to MVA85A in TB009 (Spearman’s rank correlation). C. Surface expression of TLR1 varies greatly between individuals. D. Median fluorescence intensity (MFI) of surface TLR1 on all live cells in unstimulated day 0 samples correlates with immunogenicity (TB022).

For each of the three genes, we also performed linear regression analysis with ELISpot AUC values for individual volunteers. This analysis is not possible with a high number of genes but could be informative when thinking of the mechanisms linking vaccine sensing and immunogenicity. The levels of TLR1 mRNA in both trials correlated with AUC (Figure 4a; p < 0.05, Spearman’s r=0.70, p<0.01, Spearman’s r=0.55 respectively).

We examined the original 24 samples by flow cytometry, gating on live cells, and looked at mean fluorescence intensity of TLR1. TLR1 is ubiquitously expressed and, as the original microarray analysis was done on PBMC, we did not narrow down the population further using other markers. There was a correlation between the level of TLR1 expression on day of vaccination and AUC, although the relationship was weaker at the protein level compared to the mRNA (Figure 4b,c; p<0.05, Spearman’s r=0.45).

### Blocking signalling through the TLR2 axis reduces responses to MVA85A *in vitro*

**Figure 5:**
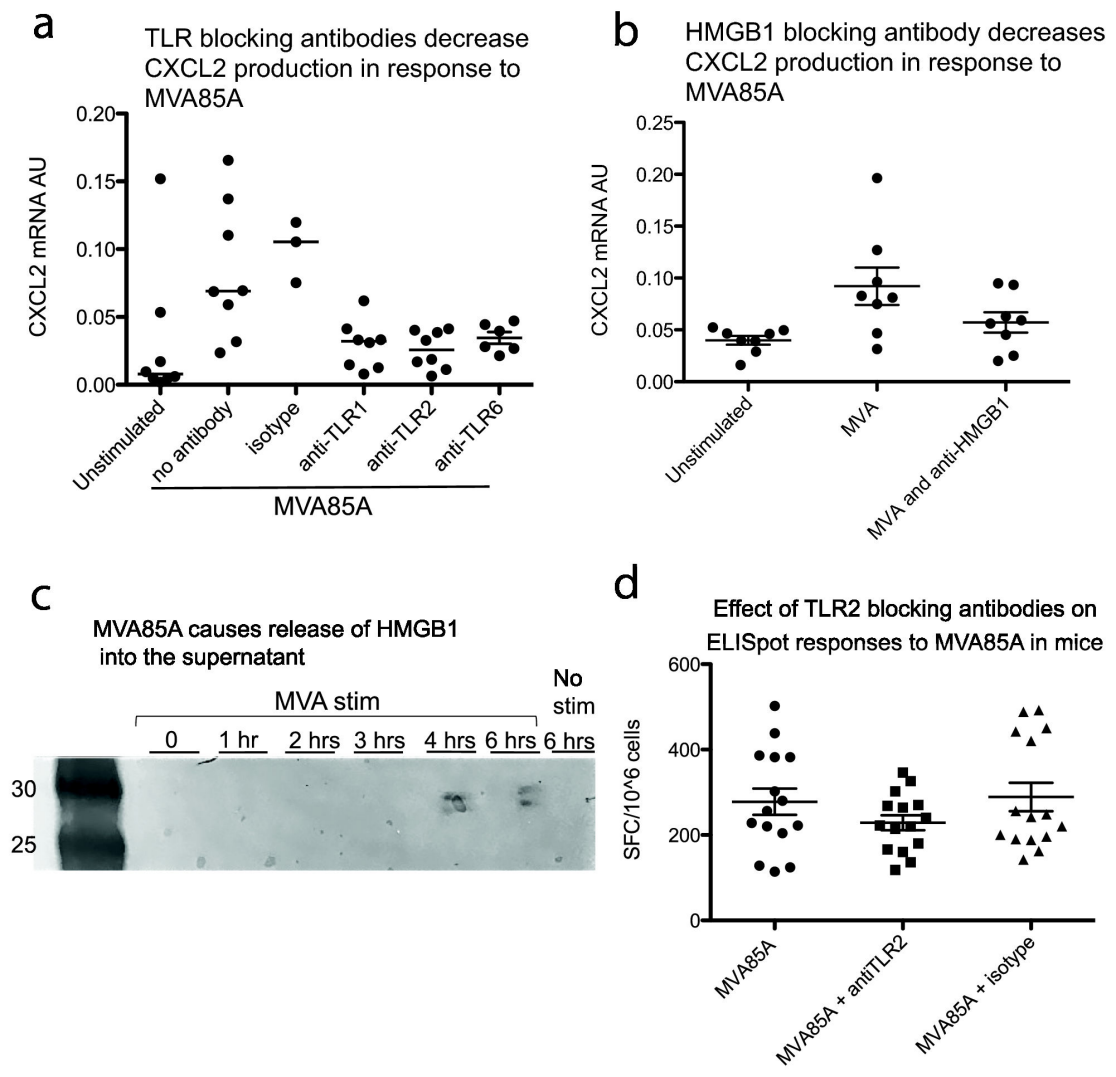
Toll-like receptors and HMGB1 in the innate response to MVA85A. A. Incubating PBMC with TLR blocking antibodies decreases CXCL2 production in response to MVA85A (Mann-Witney test for all 4 comparisons 0.01<p<0.05). B. Anti-HMGB1 also reduces CXCL2 production in response to MVA85A stimulation (Wilcoxon paired test, p<0.05). C. Western blot showing HMGB1 is released into the supernatant of MVA85A stimulated but not unstimulated cells. D. Mixing anti-TLR2 to MVA85A when vaccinating mice reduces the spread of responses (F-test, p<0.05) but not the mean (student’s t-test, p=0.18 MVA vs anti-TLR2, p=0.12 MVA85A vs isotype control).

Following analysis of the microarray data, we sought to examine the relationship between the innate response to MVA85A and TLR1 *in vitro*. TLR1 and TLR6 are located in tandem on chromosome 4 and evolved following a gene duplication event. The two receptors share extensive homology and both signal as a heterodimer with TLR2 [37]. Contrary to TLR1, TLR6 is not ubiquitously expressed [38] and this may make it less likely to be identified in a microarray analysis of circulating PBMC. Due to this, and previous results showing that MVA signals through TLR2-6 rather than TLR1-2, we investigated all three receptors in the TLR2 family. Blocking signaling through all three TLRs during in vitro stimulation of PBMC with MVA85A reduces expression of CXCL2 in response to the virus (Figure 5a). MVA leads to activation of multiple innate signaling pathways. This data is consistent with previous results showing that activation of the NFκB pathway in response to MVA stimulation (in this case MVA85A) is dependent on signaling through TLR2. In contrast to previous data, we did not find a difference between blocking signaling through TLR1 or TLR6, however we found the effect of TLR1 blocking to be donor dependent (i.e. in some donors, TLR1 neutralising antibodies consistently decreased CXCL2 expression in response to MVA85A stimulation whereas with other donors there was no effect, data not shown).

### HMGB1 is secreted following MVA85A and neutralizing it reduces the response to MVA

The nuclear protein HMGB1 is released under some conditions of cell death and can signal as a DAMP through TLR2 [39–42]. It has been reported to be important in signaling following poxvirus infection [43,44] so we investigated whether it could be playing a role in this setting. HMGB1 was not one of the genes identified in the microarray, however its mechanism of action in this setting is due to a change in cellular location (ie from the nucleus of dying cells to the extracellular space where its presence acts as a danger signal) rather than an increase in transcription so this is not necessarily unexpected. HMGB1 is detectable by western blot in the supernatant of infected cells 4 and 6 hours post infection but not in the supernatant of uninfected cells after 6 hours. Furthermore, anti-HMGB1 antibodies also reduce the production of CXCL2 in cells stimulated with MVA85A (Figure 5b, c).

### Blocking signalling through the TLR2 axis reduces responses to MVA85A *in vivo*

Mice were vaccinated with 5 x 10^5^ pfu MVA85A with or without a TLR2 neutralising antibody or an isotype control. Mice were culled seven days later and responses to the antigen 85A insert determined by IFN-γ ELISpot on splenocyte responses to a 66 peptide pool. The results show a decrease in high responses to antigen 85A. The mean responses are slightly lower than in the control and isotype groups however the main difference seems to be in the spread of the distribution, particularly in the lack of high responses in the group receiving anti-TLR2 antibodies with the vaccine (Figure 5d; all three groups are normally distributed by the D’Agostino and Pearson normality test, F test for differences in variance: p=0.04 between MVA85A and anti-TLR2, p=0.02 between anti-TLR2 and isotype groups).

## Discussion

As efforts to find a successful vaccine against TB, malaria, HIV and other diseases continue to grow, there is a need to develop our understanding of the molecular and immunological events associated with different methods of vaccination. A number of characteristics make recombinant poxviruses attractive targets for vector development and there have now been many vaccine trials conducted with recombinant poxviruses expressing different antigens. These vaccines consistently induce strong Th1 responses [14,16,45] however the magnitude of these responses varies between individuals and populations with potential impact for the applicability of early clinical studies. For example, lack of enhanced efficacy in a recent trial in BCG-vaccinated infants may link to lower immunogenicity in this population compared to UK adults [6,46]. An understanding of the processes linking recognition of the vector and early immune events at a molecular level to the vaccine-elicited immune response will aid the future development of these vaccines.

In this study we begin by describing the transcriptional changes that occur *in vivo* following vaccination of healthy BCG-vaccinated UK adults with MVA85A. Expression levels of a huge number of genes are modulated by MVA85A and changes are strongly apparent in circulating PBMC two days after vaccination, reflecting a systemic activation of inflammatory pathways. The peak adaptive response to vaccination with MVA85A is relatively early at 7 days and, consistent with this, the changes in gene expression following vaccination with MVA85A are both early and transient. This is in contrast to data published on the yellow fever vaccine, which shows a peak in expression of innate genes 7 days post-vaccination. This difference likely reflects the non-replicating nature of the MVA vector [25,47]. The much earlier peak in innate signaling is likely to impact the development of the adaptive immune response. The changes in gene expression observed in the PBMC reflect the extent of the adjuvanting properties of the MVA vector as few, if any, circulating leukocytes will have been directly exposed to MVA.

Differences between differentially expressed sets of genes in the *in vivo* and *in vitro* experiments may have multiple causes. Timing will account for some differences. Additionally, contrary to circulating PBMC, most cells in the *in vitro* experiment will have been either infected with MVA or in close contact with infected cells. Changes in processes including the lysosome, apoptosis, and phagocytosis, all of which are known to be important during infection with MVA [23,47,48], are therefore visible. A large overlap in genes modulated by both MVAwt and MVA85A in stimulated PBMC suggests the findings of this study will be applicable to other uses of MVA as a boosting vector.

Poxviruses have been selected as vaccine vectors for their ability to accommodate large inserts into their genomes (>25kb) and to induce potent T cell responses [44]. However the molecular mechanisms by which these responses are elicited, and the reason for the range of immunogenicity, are still not well understood. Heterogeneity in responses to vaccination is common and the reasons underlying it are specific to each vaccine. For example a polymorphism in TLR1 is associated with lower antibody levels in response to vaccination against *Borrelia burgdorferi* (genotyping of the reported SNP was performed in these volunteers but was not related to immunogenicity or mRNA levels, data not shown) [49]. More recently, Querec et al. demonstrated that a gene signature including the transcription initiation factor EIF2AK4 could predict antigen-specific CD8+ T cell responses to the yellow fever vaccine. This kinase, also known as GCN2, can activate the integrated stress response secondary to arginine depletion and the authors speculate that induction of the stress response directs the adaptive response to vaccination in this setting [25].

Two days after vaccination, low responders had higher expression of genes associated with the regulatory response, notably CTLA4, IL2RΑ (CD25) and STAT5B. CTLA4 is found on the surface of T cells and mediates inhibitory signals through binding of CD80 and CD86 on antigen presenting cells (APCs), IL2RΑ is a surface marker of Tregs and STAT5B regulates transcription of a number of Treg associated genes. Patients with a deficiency in the STAT5B pathway have been shown to have fewer circulating Tregs. Expression of these genes correlated with decreased responses to MVA85A. Furthermore, these early changes in gene expression reflected a difference in the expansion of Tregs, which was seen in low but not high volunteers. Low expression of STAT5B on day 0 and CTLA4 on day 2 both showed a weak correlation with Treg numbers on day 7. These associations suggest that Tregs are an important contributor in determining the magnitude of the IFN-γ ELISpot response. Although drawing firm conclusions from small data sets is difficult, these preliminary data warrant further investigation into the role of Tregs and Treg markers in response to vaccination with MVA vectored vaccines. This increase in regulatory activity in low responders is consistent with previous work showing that high levels of TGF-β in the serum on day of vaccination with MVA85A resulted in lower IFN-γ ELISpot responses post-vaccination [50]. Although we saw a difference in the Treg population, we do not know what factors are causing a stronger regulatory response to develop in some vaccinees but not others, or whether vaccination is simply accentuating differences in the Treg compartment that were already present in volunteers. Both genetic and environmental factors are likely to influence the regulatory response to MVA85A, which may be specific to the vaccine or a reflection of the immune status of an individual at the time of vaccination. Additionally, we cannot exclude the possibility that expression of TLRs and regulatory receptors may be linked.

Expression levels of three genes on day of vaccination, all involved in pattern recognition and triggering of an innate signal, allow good classification of high and low responders in two different trials. This suggests that the number of certain pattern recognition receptors (PRRs) at the time of vaccination strongly influences the number of antigen-specific IFN-γ secreting cells that develop. In particular, TLR1 expression correlates with immunogenicity, leading us to hypothesize that MVA signaling through the TLR1-2-6 axis is important in the development of the adaptive immune response. *In vitro* work shows that innate signaling through NF-κB is decreased following stimulation with MVA85A in the presence of neutralizing antibodies to TLR1, 2 or 6. Additionally, blocking signaling through these receptors by including a TLR2 antibody with the MVA85A vaccine administered to mice also appears to diminish high ELISpot responses, although the mean response to vaccination is not significantly lower. This suggests that the strength of the signal through TLR2 is affecting the magnitude of the adaptive response that develops but it is neither the only innate mechanism by which MVA is sensed nor is it the sole determinant of antigen specific immunity. Poxviruses have been infecting human populations for millennia and elicit a plethora of innate responses [44]. The strength and versatility of the innate response to MVA is evident from the array data and has been shown by other groups [51]; although it is dominated by interferon responses, different aspects of the innate response may be playing different roles in directing the adaptive response to vaccination.

Although MVA and its parent strain vaccinia have previously been shown to signal through TLR2 [23,52], no viral ligand for this interaction has been proposed and TLR2 is better known for binding to molecular patterns of bacterial and fungal origin [53]. HMGB1 has been suggested as a ligand for TLR2 in signaling following infection with different poxviruses as it is released during cell death and many attenuated strains of vaccinia virus cause widespread cell death, having lost molecules which inhibit apoptosis in the parent strain [43,54]. We show that HMGB1 is released from cells following MVA85A stimulation and that antibodies to HMGB1 can decrease CXCL2 expression in these stimulations. These data suggest that response to cell death following vaccination with MVA85A, in particular through the release of HMGB1 and its interaction with TLR2, is a strong determinant of the magnitude of the adaptive immune response that develops. Consistent with this, a recent study by Perdiguero and colleagues has shown that deletion of the anti-apoptotic gene F1L from the candidate HIV vaccine MVA-C enhances its immunogenicity in mice [22]. Furthermore, it has been shown that, in dendritic cell cultures infected with MVA, the majority of the functional response and maturation was observed in non-infected bystander cells [20]. This leads us to the model that vaccination with MVA85A causes death of infected cells and the release HMGB1, which then signals through TLR2 and either TLR1 or TLR6. The magnitude of this signal, which in humans may be determined by the considerable range of TLR expression seen on the surface of PBMC from healthy adults, is one of the factors determining the magnitude of the developing adaptive response to the vaccine. HMGB1 can under some conditions also activate TLR4 [42], which signals through the adaptor protein TICAM2. Therefore it is possible that the two pathways have different relative contributions in different individuals and thus including both can refine classification models. As with the case of the yellow fever vaccine, a danger-associated signal resulting from cellular stress or damage appears to be a strong determinant of the magnitude of the adaptive response.

In summary, we find that low responses to vaccination with MVA85A are associated with lower expression of TLR1 on day of vaccination and a regulatory response leading to expansion of Tregs over the seven days following vaccination. This study highlights features of the immune response that are important in determining the number of antigen-specific IFN-γ secreting cells in response to vaccination with MVA85A boosting BCG-induced responses. It is possible that the relative influence of different innate mechanisms on the development of the adaptive response may change when MVA is used with a different priming vaccine or if another measure of immunogenicity is investigated. However this study shows mechanisms important in humans in determining the often-measured immune parameter of antigen-specific IFN-γ secreting cells, and furthers the understanding of the relationship between early innate responses to vaccination and how these relate to the facets of the adaptive immune response that are being measured. Generating hypotheses from a human trial ensures the mechanisms being studied are relevant to the target species for vaccination, although further work will be needed to establish whether these results are applicable to other populations. Determining the importance of these mechanisms in the development of the vaccine-induced immune response in infants and HIV-infected adults in TB endemic countries will be of particular importance. Hopefully studies like these, combined with data on correlates of protective immunity emerging from recent efficacy trials, can inform the design of future vaccines.

## Materials and Methods

### Clinical trials

PBMC used in these studies were from volunteers from 2 clinical trials in which volunteers (aged 18-55) were recruited on the basis of prior BCG vaccination. The trials were registered on the clinical trials database (ClinicalTrials.gov IDs: NCT01181856 (TB022) and NCT00465465 (TB009)). Volunteers received a dose of 1 x 10^8^ pfu MVA85A injected either intradermally or intramuscularly. Both trials were fully approved by the ethical and regulatory agencies (Oxfordshire REC A and the Medicines and Healthcare products Regulatory Agency). Full written consent was obtained from each subject prior to enrollment in the trial, which was conducted according to the principles of the Declaration of Helsinki and in accordance with Good Clinical Practice (GCP). Storage of samples for exploratory immunological analyses was fully ethically approved.

### Cryopreservation of PBMC and ex-vivo IFN-γ ELISpot

PBMC from vaccinated subjects were cryopreserved in liquid nitrogen at time of acquisition as previously described [55]. IFN-γ ELISpots were performed *ex-vivo* at screening and weeks 1,2,4,12, and 24 post-vaccination as previously described [29]. The safety and primary immunological outcomes of these trials have been published elsewhere [30,36].

### Cell thawing, in vitro stimulation and RNA extraction

Frozen PBMC were thawed washed and counted as previously described [55]. 2x10^6^ cells were lysed in 350µL RLT buffer containing 10µL/mL β-mercaptoethanol and stored at -20^o^C. The RNA was extracted using the Qiagen RNeasy mini kit according to manufacturer’s instructions and stored at -80^o^C.

For the *in vitro* stimulation experiments, cells were rested 2 hours in benzonase endonuclease (Novagen), washed, then 2x10^6^ cells for each condition were stimulated for 6hrs with either media alone (10% FBS (Fetal Bovine Serum; Biosera Ltd.), 2 mM L-glutamine, 100 U/mL penicillin, 100 µg/mL streptomycin in RPMI 1640) or media containing antigen85A (20 µg/mL), wild type MVA or MVA85A both at an MOI of 1 (MOI of 5 for the blocking experiments). The stimulation volume was 200 µL. In the TLR blocking experiments, 20µg/mL of the following antibodies were added to the medium as indicated. All antibodies were neutralizing polyclonal rat IgG from ivivogen; TLR1 (pab-hstlr1), TLR2 (pab-hstlr2), TLR6 (pab-hstlr1) and isotype control (pab-sctr). The cells were then lysed and frozen in 350µL RLT buffer containing 10µL/mL β-mercaptoethanol and RNA extracted as above.

### Gene Expression Analysis

The RNA was amplified using the Illumina Totalprep RNA Amplification Kit (Ambion), according to manufacturer’s instruction. The median yield after amplification was 6.6 µg RNA (range 4-15.3µg). The quality of the RNA was checked on an Agilent bioanalyser following extraction (median RNA Integrity Number (RIN) of 8.6: range 7.4-9.7) and again following amplification. Amplified RNA (750ng per array) was hybridized to the Illumina HumanHT-12 version 4 expression beadchip according to manufacturer’s instructions. Arrays were scanned with an Illumina bead array reader confocal scanner.

### Quantitative PCR

RNA was reverse transcribed to cDNA using oligo-dT and the Omniscript reverse transcription kit (Qiagen). cDNA was stored at -20°C until qPCR was performed, using the Roche LightCycler® 480 and Quantitect mastermix (Qiagen). Quantified, purified and diluted PCR product was used to generate internal standard curves for each primer pair (table 3). Cycle number values were converted to copy number using these curves. Cycling conditions consisted of an initial activation step of 15 minute at 95°C followed by 45 cycles of 15 seconds at 94°C, 20 seconds at 60 °C and 15 seconds at 72 °C, for each primer pair.

**Table 3:** Primer sequences for quantitative PCR.

Gene	Forward primer	Reverse primer
GAPT	CCTGCCAAACTTATCCTTCCTTCACAGC	ATGCGGGTTTGGAGTGATTCAGGA
ZMYND15	TCCTGAATCACTCCAAACCCGCAT	GTGATGTTCTAGGCATTTGGGCAG
MGST1	TTGGCCTCCTGTATTCCTTGAGTG	TGGTAGATCCGTGCTCCGACAAAT
NPL	CAACTCTCAGTCATTCATTTCACAGAT	AGGATTAGGAACCAGAGACCGAGA
CD300LF	AAGAGGCCAGAAGGTCAAAGAGGT	TTTCAATGGGCTCAAGTCAAGGCG
FPR3	TGAGTCATTCCAGGATGAGTGGCT	ACACCCACAGTGGCCTCATTATCT
TLR1	AACCCATTCCGCAGTACTCCATTC	ATGGCTGCCCTTAAGTTAGCCCAA
TICAM2	AGTACCGGGATCTGCACACATCTT	AGGCTTGACTTACTTGCATGCTCC
CD14	AACTCCCTCAATCTGTCGTTCGCT	TTCAGTCTGTTGCAGCTGAGATCG
TLR6	ATATGGCTTCATGGCAGCAAGGGA	AATATGATTCACAGGGCACTCCGAGG
CXCL2	TAGCCACACTCAAGAATGGGCAGA	ACAGCCACCAATAAGCTTCCTCCT

### Flow cytometry

Anti-human antibodies (PE anti-TLR1, PB anti-CD19, AF700 anti-CD3, APC anti-CD4, APC-AF700 anti-CD8, AF647 anti-Foxp3, APC-Cy7 anti-CD25, PE anti-CTLA4) were purchased from Biolegend and QDot655 anti-CD14 and the ViViD Live/Dead cell stain from Invitrogen. A FACSCalibur (Becton Dickinson) was used for flow cytometry event collection and events were analysed using FlowJo (Tree Star Inc.). The gating strategy is shown in Figure S3.

### Western blot

Stimulations with MVAwt were stopped after 0 minutes, 30 minutes and 1, 2, 4 and 6 hours and the supernatants stored at -20 ^o^C. They were then thawed and concentrated for 20 minutes in Amicon Ultra-0.5 10kDA filters (Millipore UK) according to the manufacturer’s instructions. The supernatant was mixed 1:1 with loading dye, run 1 hour at 100 Volts on a 4-20% precise protein gel (Thermo Scientific Pierce) and transferred to a membrane (Trans-Blot Turbo Transfer Pack, Bio-Rad Laboratories Ltd) by Turbo blotting for 7 minutes. The membrane was incubated sequentially for 1 hour with PBS with 3% BSA alone, primary antibody (mouse anti-human HMGB1, Biolegend, UK) and finally secondary antibody (Alkaline Phosphatase-AffiniPure Donkey Anti-Mouse IgG, Stratech Scientific Limited). The membrane was washed and developed. Unfortunately a good loading control for western blots using cell supernatants has not been published and is not routinely used [40,56].

### Mouse experiments

All experiments were done on 6- to 8-wk-old female BALB/c mice (Harlan Orlac). All procedures were carried out under the terms of the UK Animals (Scientific Procedures) Act Home Office Project Licence (UK Home Office PPL 30/2412) and were approved by the University of Oxford Animal Care and Ethical Review Committee. Mice were vaccinated intradermally (i.d.) in each ear (25 µl/ear). Groups of 5 mice were vaccinated with 5×10^5^ plaque forming units (pfu) of MVA85A (MVA) in PBS alone or also containing 20µg of anti-TLR2 antibody (MAB 1530, R&D Systems) or an isotype control. The mice were culled 7 days later and ammonium chloride lysis buffer (ACK)-treated splenocytes used in an IFN-γ ELISpot assay. The cells were stimulated with either media alone, PHA and PMA or antigen 85A 66 peptide pool as previously described [57].

### Microarray Data Processing

Raw illumina probe data was exported from Beadstudio and screened for quality. Gene expression data was analysed using the bioconductor platform in R [58]. Genes not expressed above background levels in any sample were removed (p<0.05). In limma [59,60], background correction and quantile normalization was done using the neqc function [61]. Probes with an interquartile intensity range <0.3 (log_2_ transformed) across all samples were filtered using bioconductor’s genefilter package. The remaining 22000 probes were retained for differential expression analysis. Using limma, fold-change in expression between predefined groups was estimated. p-values from the resulting comparison were adjusted for multiple testing according to the method of Benjamini and Hochberg set to 0.05. Array quality was accounted for in the analysis using limma’s arrayWeights function, which estimates the relative reliability of each array by measuring how well its expression values follow the linear model.

Pathway analysis was performed using two web-based tools; DAVID (Database for Annotation, Visualisation and Integrated Discovery) and GSEA (Gene Set Enrichment Analysis). These two methods analyse upregulated genes by grouping them into pathways and transcriptional modules, which allows greater analytical power and biological insight. Whilst DAVID relies on published pathways and gene ontology databases to generate results, GSEA uses published gene sets. In GSEA, lists of differentially expressed genes were ranked by fold-change in expression and used in the preranked analysis. We systematically tested gene sets from the Molecular signature Database (MsigDB, http://www.broad.mit.edu/gsea/msigdb) C2 collection collected from various sources such as online pathway databases and publications in PubMed.

To see whether any genes could predict the immune response to vaccination, we used a nearest shrunken centroid method in the R package prediction analysis for microarrays (pamR). The gene list was first filtered to exclude any gene whose fold change in gene expression was less than 1.4 (2^0.5^) in at least 40% of volunteers following vaccination, leaving approximately 5000 genes. Through an interactive interface, pamR uses a nearest shrunken centroid method to make an estimate of cross-validation error for each threshold value and the optimal threshold yielding the lowest cross-validation error rate chosen. In this study, this resulted in the selection of 9 genes that could classify the vaccines with a maximal efficacy of 87%. These were taken forwarded for a validation study using quantitative PCR.

### Statistical analyses

To obtain a single measure of immunogenicity, an area under the curve analysis (AUC) was done in Prism on ELISpot immunogenicity data for each volunteer. The time points used were screening (as the baseline) and 1, 2, 4, 12 and 24 weeks post-vaccination. Volunteers were classed as “high” or “low” responders based on whether their ELISpot AUC was above or below the median (41,000 SFU.weeks/10^6^ cells in both trials).

For the real-time RT-PCR data the mean *C*t value of duplicate capillaries was converted to copy number using internal standard curves generated from purified and quantified PCR product. Expression levels for each gene were normalised by dividing copy number of gene by copy number of the housekeeping gene HPRT. For TLR1, normalized copy number was correlated to AUC using Spearman’s rank test in Prism. This test was also used to correlate AUC to TLR1 levels measured by flow cytometry. Also using GraphPad Prism, the student’s t-test was used to compare means in the *in vitro* stimulations, D’Agostino and Pearson’s test to check for normality and the F-test to compare variances.

Univariate logistic regression was done in R for each gene in the validation trial. Those genes with moderate discriminatory power (p≤0.2) were further analysed using linear discriminant analysis (LDA) in the R package MASS. LDA discriminates between two or more classes by maximizing the ratio between class variance and within class variance using a linear combination of input variables. Once a model is built, Euclidean distance is used to classify new samples. The LDA Classification error was assessed using cross validation.

### Accession code

Gene Expression omnibus, microarray data: GSE40719.

## Supporting Information

Figure S1 Boxplot showing IFN-γ ELISpot responses to antigen 85A peptide pools for the two trials from which samples in this study were taken. TB022 (open bars) and TB009 (shaded bars). Plot shows median, interquartile range and maximum and minimum. Primary data has been published:
TB022:
Meyer J, Harris S, Satti I, Poulton ID, Poyntz HC, et al. (2013) Comparing the safety and immunogenicity of a candidate TB vaccine MVA85A administered by intramuscular and intradermal delivery. Vaccine 31: 1026–1033.
TB009:
Pathan A, Minassian AM, Sander CR, Rowland R, Porter DW, et al. (2012) Effect of vaccine dose on the safety and immunogenicity of a candidate TB vaccine, MVA85A, in BCG vaccinated UK adults. Vaccine: 1–9.
(EPS)

Figure S2 Heatmap of individual samples using a list of differentially expressed genes between days 0 and 2 (a. fdr corrected p<0.05; b. fdr corrected p<0.0005). Function uses complete linkage clustering of euclidean distance. (TIFF)

Figure S3 Samples for flow cytometry analysis of Tregs or TLR1 were gated as shown above for steps 1-3: cells (FSC-A, SSC-A), singlets (FSC-A, FSC-H). Dump: ViVid and CD19 on Pacific blue. For Treg analysis cells were selected as shown: CD14-, CD3+, CD4+ and CD25+ Foxp3+. For analysis of TLR1 expression, MFI of TLR1-PE was calculated after gating out of ViVid dead and CD19+ cells as shown in Figure 3.
(EPS)

Table S1 Differentially expressed genes between *in vitro* stimulated PBMC. Differentially expressed genes between MVA85A (up in bold) and MVA wild type (up in italics) stimulated PBMC. Fdr<0.05.
Venn diagram showing overlap of deifferentially expressed genes between stimulation with antigen 85A, MVAwt or MVA85A, all compared to negative control (media only).
List of genes differentially expressed by all three stimulations compared to controls.
We thank the volunteers who took part in the study and the Oxford Genomics Group at the Wellcome Trust Centre for Human Genetics.
(DOCX)
