# Supplementary figures and images for: Roles for Treg Expansion and HMGB1 Signaling through the TLR1-2-6 Axis in Determining the Magnitude of the Antigen-Specific Immune Response to MVA85A
Source: PLoS One. 2013 Jul 3;8(7):e67922. doi: 10.1371/journal.pone.0067922 (PMC3700883; doi:10.1371/journal.pone.0067922)

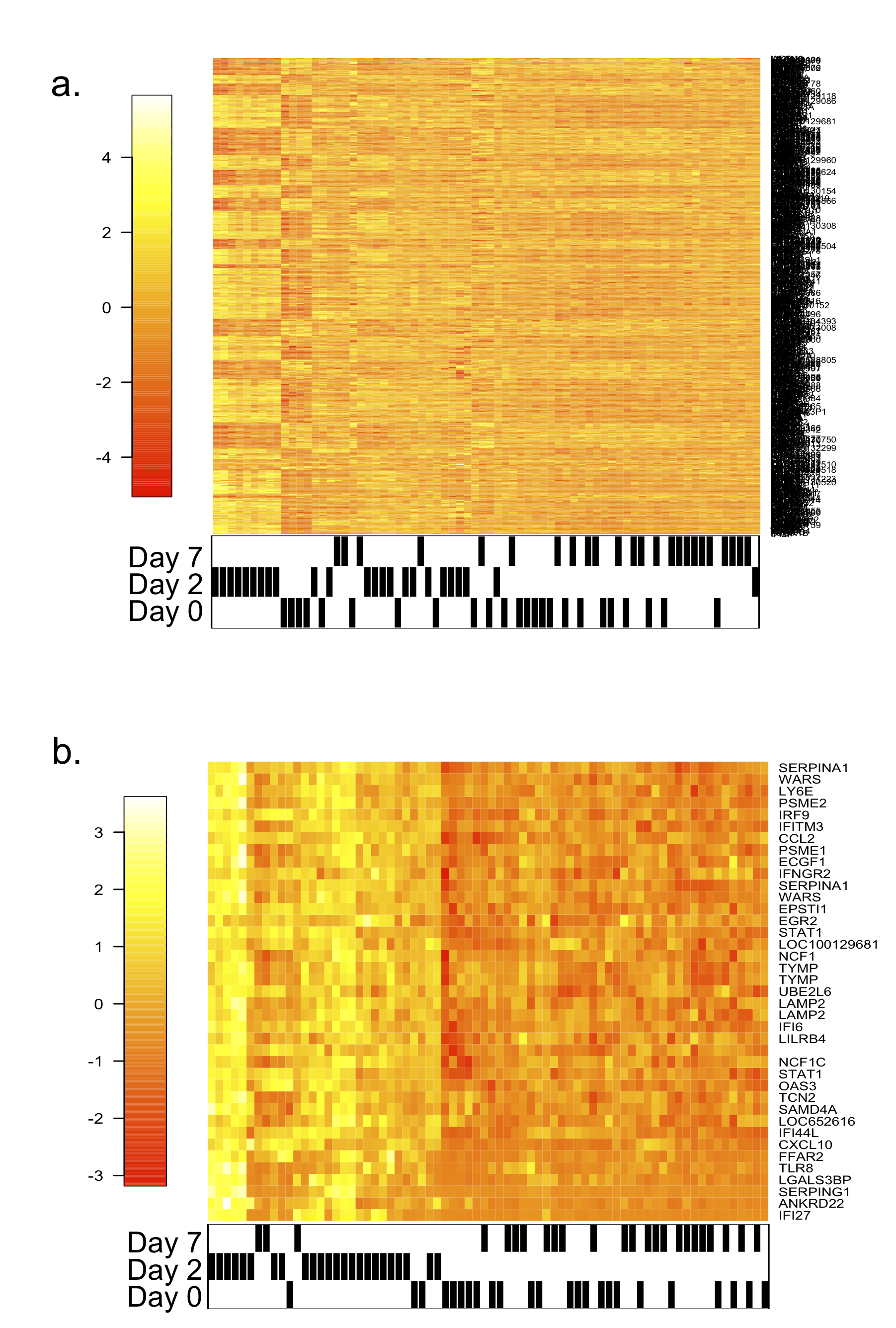

Supplement: Figure S2 — (TIFF) [file pone.0067922.s002.tiff]
