# Supplementary material for: Roles for Treg Expansion and HMGB1 Signaling through the TLR1-2-6 Axis in Determining the Magnitude of the Antigen-Specific Immune Response to MVA85A
Source: PLoS One. 2013 Jul 3;8(7):e67922. doi: 10.1371/journal.pone.0067922 (PMC3700883; doi:10.1371/journal.pone.0067922)
Supplement: Table S1 — Differentially expressed genes between MVA85A (up in bold) and MVA wild type (up in italics) stimulated PBMC. Fdr<0.05. Venn diagram showing overlap of deifferentially expressed genes between stimulation with antigen 85A, MVAwt or MVA85A, all compared to negative control (media only). List of genes differentially expressed by all three stimulations compared to controls. We thank the volunteers who took part in the study and the Oxford Genomics Group at the Wellcome Trust Centre for Human Genetics. (DOCX) [file pone.0067922.s004.docx]

| The table below shows differentially expressed genes between *in vitro* stimulation with MVA85A and wild type MVA | | | | |  |
| --- | --- | --- | --- | --- | --- |
| **bold:** higher expression following MVA85A | | | | |  |
| *italic*: higher expression following wild type MVA | | | | |  |
| SYMBOL | logFC | AveExpr | adj.P.Val | fold change |  |
| **CCL2** | 3.199371637 | 10.19477087 | 0.001232371 | 9.185585203 |  |
| **AQP9** | 3.173484524 | 9.476314605 | 2.95E-05 | 9.022232871 |  |
| **KLF4** | 2.747707002 | 6.137951016 | 0.003158233 | 6.71648775 |  |
| **LSM12** | 2.552876891 | 6.762975052 | 7.65E-06 | 5.868032621 |  |
| **MTMR11** | 2.438498532 | 7.151881161 | 0.000988638 | 5.420772766 |  |
| **SRC** | 2.430184134 | 9.105663871 | 0.001611 | 5.389622151 |  |
| **IL1RN** | 2.333492521 | 8.61274866 | 0.000495983 | 5.040240311 |  |
| **ZC3H12C** | 2.297173814 | 7.069296589 | 0.00058323 | 4.914940031 |  |
| **LOC643930** | 2.265203152 | 5.830502062 | 0.00585032 | 4.807221076 |  |
| **CXCL1** | 2.201266101 | 8.943823157 | 0.028902289 | 4.598827556 |  |
| **BCL11A** | 2.198113333 | 6.519579809 | 0.005935229 | 4.588788563 |  |
| **ANPEP** | 2.175286213 | 8.794124336 | 0.000471887 | 4.516753598 |  |
| **PVRL2** | 2.11532103 | 7.180149264 | 0.000495983 | 4.332864228 |  |
| **DNAJB5** | 2.081729982 | 6.643372735 | 0.00089404 | 4.23314522 |  |
| **RHOU** | 2.048511248 | 8.145549885 | 0.016994942 | 4.136788638 |  |
| **LINCR** | 2.019825986 | 6.051251834 | 0.025268447 | 4.055348745 |  |
| **ADAP1** | 2.018741348 | 6.659560482 | 0.000471887 | 4.052301022 |  |
| **ITGAX** | 1.999270508 | 8.726849215 | 0.012634844 | 3.997977929 |  |
| **C14orf4** | 1.982262263 | 8.267314241 | 0.026273034 | 3.951121644 |  |
| **AP2B1** | 1.982011392 | 6.302993751 | 0.000214819 | 3.950434639 |  |
| **KLF4** | 1.976001688 | 6.143557862 | 0.007768271 | 3.934012899 |  |
| **BCL11A** | 1.975193012 | 7.425380972 | 0.021061347 | 3.931808378 |  |
| **PLEKHA4** | 1.968289379 | 7.008133639 | 0.00115188 | 3.913038701 |  |
| **SLC43A3** | 1.951534115 | 8.667954445 | 0.011019671 | 3.867856083 |  |
| **TGFBI** | 1.945844457 | 9.047357906 | 0.014712803 | 3.852632185 |  |
| **TAGLN** | 1.939385687 | 6.461870437 | 0.015483834 | 3.835422969 |  |
| **LOC90925** | 1.930530094 | 6.67946442 | 0.016799071 | 3.811952372 |  |
| **FGR** | 1.926441818 | 8.490498016 | 0.001665324 | 3.801165441 |  |
| **RRAD** | 1.88973567 | 5.304038984 | 0.043342307 | 3.705673233 |  |
| **SOD2** | 1.876595594 | 11.92520845 | 0.000988638 | 3.672075176 |  |
| **KYNU** | 1.858309098 | 11.05365935 | 0.006513247 | 3.625824505 |  |
| **EXT1** | 1.841068138 | 7.8617009 | 0.000640786 | 3.582751888 |  |
| **THBS1** | 1.829578619 | 9.498255158 | 0.011736393 | 3.554332426 |  |
| **C7orf20** | 1.796922602 | 6.83572473 | 0.022722977 | 3.474782319 |  |
| **NCF2** | 1.780471845 | 7.983728022 | 0.028773602 | 3.435385132 |  |
| **MKL1** | 1.779528267 | 7.072669434 | 0.033620292 | 3.433138994 |  |
| **P2RX4** | 1.776696139 | 7.923699232 | 0.010484271 | 3.426406072 |  |
| **PLAUR** | 1.762924206 | 8.378957047 | 0.001232371 | 3.393853301 |  |
| **RSPRY1** | 1.758372142 | 7.21330068 | 0.047212249 | 3.383161722 |  |
| **CYP1B1** | 1.732705037 | 8.866719748 | 0.010630588 | 3.323503877 |  |
| **HLX** | 1.715234154 | 6.672168221 | 0.046441057 | 3.283499315 |  |
| **SDC4** | 1.706145375 | 6.830020109 | 0.019155883 | 3.262878744 |  |
| **CD14** | 1.700909206 | 10.09299692 | 0.021725582 | 3.2510578 |  |
| **CUTL1** | 1.699350079 | 7.384576352 | 0.039600107 | 3.247546266 |  |
| **PREP** | 1.696099135 | 7.508204421 | 0.003675282 | 3.24023654 |  |
| **LOC650034** | 1.681789116 | 6.218904392 | 0.002339814 | 3.208255668 |  |
| **AK3L1** | 1.664580853 | 7.097040218 | 0.016994942 | 3.170215367 |  |
| **IL4I1** | 1.647757978 | 7.616974101 | 0.033570987 | 3.133463045 |  |
| **CTSL1** | 1.644520709 | 11.06914178 | 0.002339814 | 3.126439736 |  |
| **IFNB1** | 1.641500249 | 8.00074522 | 0.001311328 | 3.119900996 |  |
| **CTSL1** | 1.627870828 | 11.18737389 | 0.016484622 | 3.090565471 |  |
| **NUTF2** | 1.603566092 | 6.662574499 | 0.030046841 | 3.038935578 |  |
| **FAM108A3** | 1.571233486 | 6.412495461 | 0.024939169 | 2.971586724 |  |
| **RANBP3L** | 1.511765466 | 5.623605928 | 0.002163626 | 2.851587824 |  |
| **CD200** | 1.499427878 | 6.386316326 | 0.042597055 | 2.827305692 |  |
| **RMRP** | 1.481559128 | 6.651105395 | 0.02053775 | 2.792503575 |  |
| **STAT3** | 1.457929322 | 9.825530437 | 0.047163381 | 2.74713788 |  |
| **HAX1** | 1.448604939 | 6.804941515 | 0.024528412 | 2.729439916 |  |
| **FCGRT** | 1.442112812 | 7.547763276 | 0.006513247 | 2.717185032 |  |
| **ARMCX1** | 1.42442911 | 6.176489275 | 0.015483834 | 2.684082675 |  |
| **CD8B** | 1.412368068 | 6.575607353 | 0.047010737 | 2.661737071 |  |
| **LOC100133800** | 1.40879717 | 5.828669962 | 0.035146496 | 2.655156999 |  |
| **KIAA0495** | 1.400728315 | 6.428980888 | 0.012015721 | 2.640348412 |  |
| **ANXA5** | 1.394925182 | 11.24737602 | 0.003148804 | 2.629749138 |  |
| **IER3** | 1.388994931 | 11.63327026 | 0.00585032 | 2.618961643 |  |
| **SOD2** | 1.345517434 | 13.58078824 | 0.001125383 | 2.541213229 |  |
| **LOC441124** | 1.341991235 | 6.665724935 | 0.020656083 | 2.535009643 |  |
| **NOTCH2NL** | 1.331317634 | 5.597561883 | 0.002339814 | 2.516323895 |  |
| **ADM** | 1.326913856 | 9.688656081 | 0.038977935 | 2.508654611 |  |
| **NAPSB** | 1.312247743 | 8.589123567 | 0.024939169 | 2.483281381 |  |
| **C15orf48** | 1.289846613 | 11.32227926 | 0.003148804 | 2.445020587 |  |
| **LOC653994** | 1.284504317 | 5.78603186 | 0.002881303 | 2.435983426 |  |
| **OR2A14** | 1.281358289 | 5.268437566 | 0.029531833 | 2.430677161 |  |
| **AGFG2** | 1.27963923 | 5.714333457 | 0.047566576 | 2.427782586 |  |
| **LYN** | 1.261865339 | 10.58055139 | 0.039596505 | 2.398055982 |  |
| **KYNU** | 1.25533496 | 10.35381602 | 0.010630588 | 2.387225679 |  |
| **NR4A3** | 1.239683699 | 5.979720255 | 0.030046841 | 2.36146753 |  |
| **TBC1D10A** | 1.238238005 | 6.592074088 | 0.040226981 | 2.35910234 |  |
| **ITPRIPL2** | 1.231568268 | 7.200633962 | 0.002163626 | 2.348221123 |  |
| **HLX** | 1.230796172 | 6.372247629 | 0.048151893 | 2.346964748 |  |
| **PMS2CL** | 1.214831433 | 6.310340891 | 0.03487631 | 2.321136608 |  |
| **PLEK** | 1.207343833 | 10.528427 | 0.015577876 | 2.309121096 |  |
| **SPRYD3** | 1.20606316 | 6.919527802 | 0.030046841 | 2.30707221 |  |
| **RPP14** | 1.204615852 | 5.740132132 | 0.044901736 | 2.304758921 |  |
| **LOC100131336** | 1.196531292 | 5.358573914 | 0.016790761 | 2.291879656 |  |
| **LOC651694** | 1.188728729 | 5.053525809 | 0.022406212 | 2.279517886 |  |
| **EZH2** | 1.179010936 | 5.513561013 | 0.047163381 | 2.264214967 |  |
| **HIST1H4D** | 1.17300323 | 5.1499782 | 0.048624256 | 2.254805872 |  |
| **BAG3** | 1.168390318 | 8.248423314 | 0.014249195 | 2.24760781 |  |
| **CD63** | 1.146914951 | 9.616920756 | 0.046244865 | 2.214398624 |  |
| **RBM47** | 1.100504785 | 7.403217005 | 0.015789027 | 2.144297063 |  |
| **IMAA** | 1.083327886 | 5.398992402 | 0.043342307 | 2.118918188 |  |
| **OR12D3** | 1.075230007 | 4.952900232 | 0.023423238 | 2.107057971 |  |
| **PPP3R1** | 1.064899081 | 9.508655056 | 0.047212249 | 2.092023534 |  |
| **DDR1** | 1.059612983 | 6.098194558 | 0.033570987 | 2.084372294 |  |
| **OSGEP** | 1.048489475 | 7.424430282 | 0.007498233 | 2.068363105 |  |
| **C21orf70** | 1.04787008 | 5.894114966 | 0.04902208 | 2.06747528 |  |
| **ZNF395** | 1.026801601 | 10.25080978 | 0.045172009 | 2.037502178 |  |
| **MASP2** | 1.026487216 | 5.389079499 | 0.040542841 | 2.037058223 |  |
| **C10orf73** | 1.023474493 | 6.071511115 | 0.039600107 | 2.032808754 |  |
| **LOC727825** | 1.016073747 | 5.906394608 | 0.037839484 | 2.022407539 |  |
| **DTNBP1** | 0.988505324 | 5.964782227 | 0.043342307 | 1.984128309 |  |
| **KIAA0133** | 0.979240649 | 6.179254145 | 0.035146496 | 1.971427491 |  |
| **LOC100129365** | 0.977129936 | 4.898335325 | 0.022406212 | 1.968545333 |  |
| **COL11A2** | 0.958324221 | 5.248352369 | 0.014249195 | 1.943051609 |  |
| **PFTK1** | 0.948001274 | 7.093856682 | 0.048151893 | 1.929198073 |  |
| **ANP32C** | 0.940784068 | 6.280752467 | 0.045837304 | 1.919571193 |  |
| **SLC2A1** | 0.933195674 | 9.052061745 | 0.02548349 | 1.909500999 |  |
| **ABCG4** | 0.830054822 | 5.118852357 | 0.045624701 | 1.777752915 |  |
| **LOC646723** | 0.815365984 | 6.154634216 | 0.048151893 | 1.759744508 |  |
| **MFSD2** | 0.809138717 | 5.252740507 | 0.036973301 | 1.752165095 |  |
| **LYL1** | 0.79406797 | 7.610384878 | 0.033125818 | 1.733956818 |  |
| **FTL** | 0.735143948 | 13.98856598 | 0.047212249 | 1.664563547 |  |
| *TROAP* | -0.753269276 | 5.1723711 | 0.045837304 | -1.685608245 |  |
| *HELT* | -0.766506043 | 4.999277424 | 0.033620292 | -1.701144913 |  |
| *LOC652608* | -0.838475956 | 9.134837269 | 0.020013265 | -1.788160155 |  |
| *KIAA0947* | -0.843557788 | 7.917297163 | 0.02443096 | -1.794469979 |  |
| *LOC647030* | -0.844630309 | 12.2453966 | 0.030046841 | -1.795804511 |  |
| *RNASEH2B* | -0.858038194 | 8.121623842 | 0.033125818 | -1.812571862 |  |
| *GRB7* | -0.879985774 | 5.192644898 | 0.047212249 | -1.840357154 |  |
| *FLJ40453* | -0.911327829 | 5.418954544 | 0.040542841 | -1.880775732 |  |
| *LOC643224* | -0.914787822 | 5.14964643 | 0.046244865 | -1.88529178 |  |
| *SS18* | -0.927366601 | 9.460748373 | 0.045745463 | -1.901801405 |  |
| *FLJ38973* | -0.97485936 | 5.929955859 | 0.040845979 | -1.965449588 |  |
| *NCRNA00094* | -0.980492924 | 6.213129504 | 0.040063481 | -1.973139454 |  |
| *TGM5* | -0.988272621 | 5.143033698 | 0.022862622 | -1.9838083 |  |
| *LOC651772* | -1.000512828 | 6.140981328 | 0.030721429 | -2.000711057 |  |
| *PP8961* | -1.013279555 | 5.559748438 | 0.033620292 | -2.018494358 |  |
| *TSGA14* | -1.022070334 | 5.590670562 | 0.013957224 | -2.030831206 |  |
| *LOC388401* | -1.02399355 | 7.776754301 | 0.046441057 | -2.033540255 |  |
| *RANBP2* | -1.039966445 | 5.656479063 | 0.033620292 | -2.056179829 |  |
| *C7orf70* | -1.060483769 | 5.723521179 | 0.043485144 | -2.085630765 |  |
| *NARG2* | -1.062610157 | 5.753871872 | 0.025755625 | -2.088707042 |  |
| *FLJ11827* | -1.079250586 | 5.055848069 | 0.045837304 | -2.112938221 |  |
| *C7orf11* | -1.094416509 | 6.967926247 | 0.007498233 | -2.135267043 |  |
| *LOC654069* | -1.102962293 | 5.23783195 | 0.021061347 | -2.147952802 |  |
| *LOC100129543* | -1.105980943 | 6.216381276 | 0.022722977 | -2.152451817 |  |
| *MBLAC2* | -1.131203014 | 5.69445609 | 0.024939169 | -2.190413152 |  |
| *DNAL1* | -1.140986664 | 5.559434259 | 0.00397503 | -2.205317941 |  |
| *NACAP1* | -1.152717951 | 9.919557721 | 0.010334406 | -2.22332361 |  |
| *BMX* | -1.171142417 | 5.374226257 | 0.022406212 | -2.251899459 |  |
| *APPBP2* | -1.203535402 | 5.343238442 | 0.040845979 | -2.303033508 |  |
| *LMTK2* | -1.226645133 | 5.454900072 | 0.016559077 | -2.340221575 |  |
| *ZNF385D* | -1.233801213 | 5.380153445 | 0.026977429 | -2.351858419 |  |
| *CTH* | -1.256018175 | 5.293456305 | 0.007498233 | -2.388356462 |  |
| *LOC729652* | -1.263374686 | 5.31770412 | 0.048151893 | -2.40056614 |  |
| *PRIM2* | -1.270699869 | 4.952420699 | 0.048151893 | -2.412785844 |  |
| *LOC440352* | -1.349521869 | 5.378142207 | 0.046441057 | -2.548276577 |  |
| *CCAR1* | -1.378138126 | 6.082437145 | 0.041688614 | -2.599326976 |  |
| *LOC648039* | -1.381180642 | 5.172629775 | 0.048151893 | -2.604814511 |  |
| *OR4K15* | -1.411176567 | 5.286163664 | 0.033125818 | -2.65953969 |  |
| *LOC728188* | -1.434577408 | 7.785277244 | 0.010484271 | -2.703029783 |  |
| *LOC654123* | -1.4447374 | 5.708293695 | 0.020656083 | -2.722132704 |  |
| *OR14C36* | -1.483605476 | 5.389323816 | 0.010950267 | -2.796467331 |  |
| *RNY3* | -1.49129867 | 7.988883927 | 0.02053775 | -2.811419366 |  |
| *TAF8* | -1.515834232 | 5.060033329 | 0.024939169 | -2.859641377 |  |
| *AQP12B* | -1.640472085 | 6.277968732 | 0.037839484 | -3.117678332 |  |
| *KREMEN2* | -1.6789239 | 6.649210771 | 0.002339814 | -3.201890343 |  |
| *LOC100133568* | -1.72668891 | 5.539185434 | 0.001665324 | -3.30967352 |  |
| *LOC100130552* | -1.907293575 | 5.681816129 | 0.02443096 | -3.751047612 |  |
| *LOC647104* | -2.052834698 | 7.201126373 | 0.000586999 | -4.149204309 |  |
| *MIR574* | -2.209292564 | 5.695575415 | 0.030721429 | -4.624484531 |  |
| *MIR1974* | -2.30844497 | 10.17600444 | 0.013957224 | -4.953488732 |  |
| *NOC4L* | -2.316976043 | 5.790610777 | 0.035165494 | -4.982866916 |  |
|  |  |  |  |  |  |

| \| Venn Diagram showing overlap in differentially expressed genes in 3 stimulations compared to media only control. \| \| --- \| \| \|     The following 39 probes are differentially expressed as compared to negative media only control when stimulated with antigen 85A alone, MVA wild type or MVA85A | | | |
| --- | --- | --- | --- | --- |
|  |  |  |  |
|  |  |  |  |
|  |  |  |  |
| PROBE_ID | SYMBOL |  |  |
| ILMN_1763000 | ADAP2 |  |  |
| ILMN_1774447 | AMPD3 |  |  |
| ILMN_2218856 | CCL3L1 |  |  |
| ILMN_1747355 | CCL3L1 |  |  |
| ILMN_1773245 | CCL3L1 |  |  |
| ILMN_1722622 | CD163 |  |  |
| ILMN_2379599 | CD163 |  |  |
| ILMN_1784863 | CD36 |  |  |
| ILMN_1796094 | CD36 |  |  |
| ILMN_1695423 | CD9 |  |  |
| ILMN_1733998 | DHRS9 |  |  |
| ILMN_2384181 | DHRS9 |  |  |
| ILMN_1811616 | EEPD1 |  |  |
| ILMN_1740213 | ELOVL7 |  |  |
| ILMN_2146761 | FABP5 |  |  |
| ILMN_1752728 | FUCA1 |  |  |
| ILMN_1730816 | GPR162 |  |  |
| ILMN_1669317 | GPR77 |  |  |
| ILMN_1699651 | IL6 |  |  |
| ILMN_1813704 | KIAA1199 |  |  |
| ILMN_1661631 | LILRA3 |  |  |
| ILMN_3294222 | LOC100132673 |  |  |
| ILMN_3237878 | LOC100133875 |  |  |
| ILMN_3274711 | LOC392501 |  |  |
| ILMN_1656938 | LOC731486 |  |  |
| ILMN_1770610 | MERTK |  |  |
| ILMN_2138589 | MERTK |  |  |
| ILMN_1686664 | MT2A |  |  |
| ILMN_2149494 | NPL |  |  |
| ILMN_1684982 | PDK4 |  |  |
| ILMN_1800225 | PPARG |  |  |
| ILMN_1771688 | RAB7B |  |  |
| ILMN_1795183 | RNASE1 |  |  |
| ILMN_2333670 | RNASE1 |  |  |
| ILMN_2338452 | SERPINA1 |  |  |
| ILMN_1732923 | SIPA1L2 |  |  |
| ILMN_2100357 | SMPDL3A |  |  |
| ILMN_1655987 | STAB1 |  |  |
| ILMN_1707591 | TNIP3 |  |  |
